# 2-(Benzyl­carbamo­yl)nicotinic acid

**DOI:** 10.1107/S1600536813024483

**Published:** 2013-09-07

**Authors:** Yan-Cao Mao, Hao Wu, Jin-Jun Shan, Ke Yan

**Affiliations:** a College of Pharmacy, Nanjing University of Chinese Medicine, Nanjing 210023, People’s Republic of China; b Department of Applied Chemistry, Nanjing College of Chemical Technology, Nanjing 210048, People’s Republic of China; c Molecular Biology Laboratory of SATCM, First Medicine College, Nanjing University of Chinese Medicine, Nanjing 210023, People’s Republic of China; d Science and Technology Department, Nanjing University of Chinese Medicine, Nanjing 210023, People’s Republic of China

## Abstract

In the title compound, C_14_H_12_N_2_O_3_, the pyridine ring is twisted with respect to the phenyl ring and the carb­oxy­lic acid group at angles of 37.1 (5) and 8.1 (3)°, respectively; the phenyl ring forms a dihedral angle of 41.4 (1)° with the mean plane of the C—NH—C=O fragment. An intra­molecular O—H⋯O hydrogen bond occurs between the carb­oxy­lic acid and carbonyl groups. In the crystal, N—H⋯O hydrogen bonds link mol­ecules into a supra­molecular chain running along the *a*-axis direction.

## Related literature

For background to the title compound, see: Konshin *et al.* (2010 ▶). For a related structure, see: Koch *et al.* (2008 ▶).
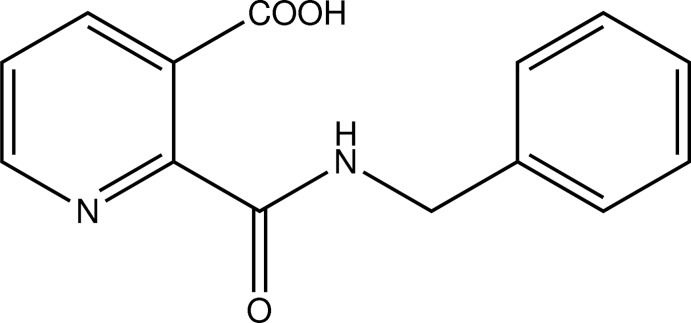


## Experimental

### 

#### Crystal data

C_14_H_12_N_2_O_3_
*M* *_r_* = 256.26
Orthorhombic, 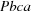

*a* = 13.024 (3) Å
*b* = 8.4110 (17) Å
*c* = 23.143 (5) Å
*V* = 2535.2 (9) Å^3^
*Z* = 8
Mo *K*α radiation
μ = 0.10 mm^−1^
*T* = 293 K
0.30 × 0.20 × 0.10 mm

#### Data collection

Enraf–Nonius CAD-4 diffractometer
4586 measured reflections
2326 independent reflections
1242 reflections with *I* > 2σ(*I*)
*R*_int_ = 0.096
3 standard reflections every 200 reflections intensity decay: 1%

#### Refinement

*R*[*F* ^2^ > 2σ(*F* ^2^)] = 0.057
*wR*(*F* ^2^) = 0.167
*S* = 1.00
2326 reflections
172 parameters
H-atom parameters constrained
Δρ_max_ = 0.22 e Å^−3^
Δρ_min_ = −0.14 e Å^−3^

### 

Data collection: *CAD-4 EXPRESS* (Enraf–Nonius, 1994 ▶); cell refinement: *CAD-4 EXPRESS*; data reduction: *XCAD4* (Harms & Wocadlo,1995 ▶); program(s) used to solve structure: *SHELXTL* (Sheldrick, 2008 ▶); program(s) used to refine structure: *SHELXTL*; molecular graphics: *SHELXTL*; software used to prepare material for publication: *SHELXTL*.

## Supplementary Material

Crystal structure: contains datablock(s) I, New_Global_Publ_Block. DOI: 10.1107/S1600536813024483/xu5724sup1.cif

Structure factors: contains datablock(s) I. DOI: 10.1107/S1600536813024483/xu5724Isup2.hkl

Supplementary material file. DOI: 10.1107/S1600536813024483/xu5724Isup3.cml

Additional supplementary materials:  crystallographic information; 3D view; checkCIF report

## Figures and Tables

**Table 1:** Hydrogen-bond geometry (Å, °)

*D*—H⋯*A*	*D*—H	H⋯*A*	*D*⋯*A*	*D*—H⋯*A*
N1—H1*A*⋯O3^i^	0.86	2.24	3.033 (3)	154
O3—H3*B*⋯O1	0.82	1.62	2.435 (3)	179

Symmetry code: (i) 
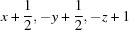
.

**Fig. 1.**
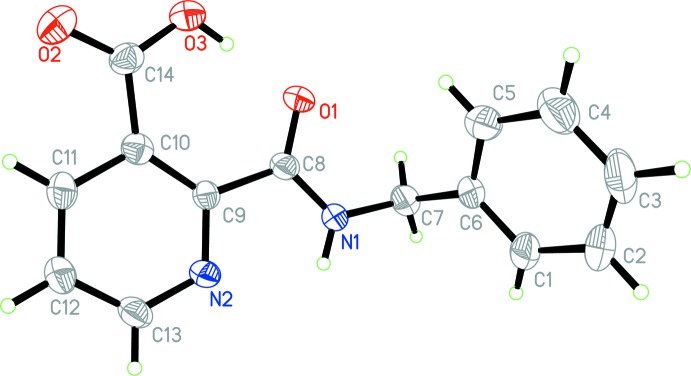
The molecular structure of (I), showing the atom-numbering scheme and displacement ellipsoids at the 30% probability level.

**Fig. 2.**
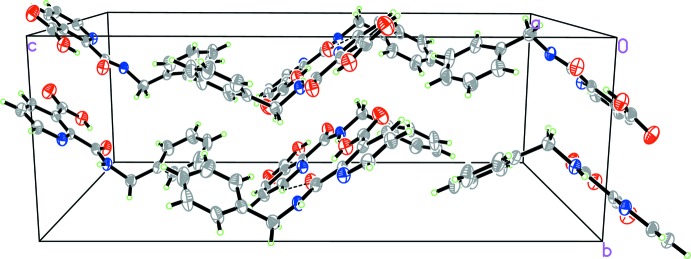
A packing diagram of (I). Intermolecular hydrogen bonds are shown as dashed lines.

## supplementary crystallographic information

### 1. Comment

Drugs that reduce blood coagulation and are widely used for therapy and prevention in surgical operations and for ischemic heart disease and other diseases are known to have several serious shortcomings (Konshin *et al.*, 2010). Our research on biologically active amides and hydrazides of pyridinecarboxylic acids led to the synthesis of substituted 3-carboxypicolinic acid amides.

The molecular structure of the title compound is shown in Fig. 1. The pyridine ring is twisted by 37.1 (5) and 8.1 (3)°, with respect to the benzene ring and carboxyl group; the benzene ring forms a dihedral angle of 41.4 (1)° with the mean plane of the C—NH—C═O fragment.

As shown in Figure 2, the molecules are linked by N—H···O hydrogen bonds into chain in the crystal lattice (Table 1).

### 2. Experimental

A solution of quinolinic acid anhydride (20 mmol) in CHCl_3_ (20 ml) was treated with Et_3_N (20 mmol). Then a solution of phenylmethanamine (20 mmol) in CHCl_3_ (15 ml) was added gradually at a rate such that the temperature of the mixture did not rise above 303 K. The mixture was left for 12 h. The resulting precipitate was filtered off, dissolved in the minimum amount of water, and precipitated by acetic acid. The precipitate was separated, washed with water and recrystallized from EtOH.

### 3. Refinement

H atoms were positioned geometrically, with O—H = 0.82 Å and N—H = 0.86 Å and C—H = 0.93 and 0.97 Å for aromatic and methine H, respectively, and constrained to ride on their parent atoms, with *U*_iso_(H) = xU_eq_(C,N,O), where *x* =1.5 for carboxyl H, and *x* = 1.2 for all other H atoms.

### Crystal data

**Table:**

C_14_H_12_N_2_O_3_	*F*(000) = 1072
*M**_r_* = 256.26	*D*_x_ = 1.343 Mg m^−^^3^
Orthorhombic, *P**b**c**a*	Mo *K*α radiation, λ = 0.71073 Å
Hall symbol: -P 2ac 2ab	Cell parameters from 25 reflections
*a* = 13.024 (3) Å	θ = 10–13°
*b* = 8.4110 (17) Å	µ = 0.10 mm^−^^1^
*c* = 23.143 (5) Å	*T* = 293 K
*V* = 2535.2 (9) Å^3^	Block, colorless
*Z* = 8	0.30 × 0.20 × 0.10 mm

### Data collection

**Table:**

Enraf–Nonius CAD-4 diffractometer	*R*_int_ = 0.096
Radiation source: fine-focus sealed tube	θ_max_ = 25.4°, θ_min_ = 1.8°
Graphite monochromator	*h* = 0→15
ω/2θ scans	*k* = 0→10
4586 measured reflections	*l* = −27→27
2326 independent reflections	3 standard reflections every 200 reflections
1242 reflections with *I* > 2σ(*I*)	intensity decay: 1%

### Refinement

**Table:**

Refinement on *F*^2^	Primary atom site location: structure-invariant direct methods
Least-squares matrix: full	Secondary atom site location: difference Fourier map
*R*[*F*^2^ > 2σ(*F*^2^)] = 0.057	Hydrogen site location: inferred from neighbouring sites
*wR*(*F*^2^) = 0.167	H-atom parameters constrained
*S* = 1.00	*w* = 1/[σ^2^(*F*_o_^2^) + (0.053*P*)^2^] where *P* = (*F*_o_^2^ + 2*F*_c_^2^)/3
2326 reflections	(Δ/σ)_max_ < 0.001
172 parameters	Δρ_max_ = 0.22 e Å^−^^3^
0 restraints	Δρ_min_ = −0.14 e Å^−^^3^

### Special details

**Table:**

Geometry. All e.s.d.'s (except the e.s.d. in the dihedral angle between two l.s. planes) are estimated using the full covariance matrix. The cell e.s.d.'s are taken into account individually in the estimation of e.s.d.'s in distances, angles and torsion angles; correlations between e.s.d.'s in cell parameters are only used when they are defined by crystal symmetry. An approximate (isotropic) treatment of cell e.s.d.'s is used for estimating e.s.d.'s involving l.s. planes.
Refinement. Refinement of *F*^2^ against ALL reflections. The weighted *R*-factor *wR* and goodness of fit *S* are based on *F*^2^, conventional *R*-factors *R* are based on *F*, with *F* set to zero for negative *F*^2^. The threshold expression of *F*^2^ > σ(*F*^2^) is used only for calculating *R*-factors(gt) *etc*. and is not relevant to the choice of reflections for refinement. *R*-factors based on *F*^2^ are statistically about twice as large as those based on *F*, and *R*- factors based on ALL data will be even larger.

### Fractional atomic coordinates and isotropic or equivalent isotropic displacement parameters (Å^2^)

**Table:**

	*x*	*y*	*z*	*U*_iso_*/*U*_eq_	
N1	0.42068 (17)	0.3451 (3)	0.54279 (10)	0.0496 (7)	
H1A	0.4839	0.3212	0.5365	0.060*	
O1	0.25706 (15)	0.3073 (3)	0.51797 (9)	0.0653 (7)	
C1	0.4634 (3)	0.3853 (5)	0.68730 (14)	0.0751 (11)	
H1B	0.5234	0.4402	0.6784	0.090*	
N2	0.49338 (17)	0.1694 (3)	0.46226 (10)	0.0514 (7)	
O2	0.17921 (17)	−0.0277 (3)	0.38457 (12)	0.0857 (8)	
C2	0.4535 (3)	0.3113 (6)	0.74105 (17)	0.0989 (15)	
H2B	0.5069	0.3171	0.7677	0.119*	
O3	0.15332 (14)	0.1519 (3)	0.45110 (10)	0.0694 (7)	
H3B	0.1874	0.2050	0.4738	0.104*	
C3	0.3665 (3)	0.2309 (6)	0.75455 (17)	0.0930 (13)	
H3A	0.3598	0.1830	0.7906	0.112*	
C4	0.2891 (3)	0.2204 (6)	0.71546 (18)	0.0911 (13)	
H4A	0.2297	0.1646	0.7249	0.109*	
C5	0.2975 (3)	0.2918 (5)	0.66178 (15)	0.0754 (11)	
H5A	0.2444	0.2824	0.6351	0.090*	
C6	0.3857 (2)	0.3779 (4)	0.64753 (13)	0.0538 (8)	
C7	0.3974 (3)	0.4559 (4)	0.58954 (13)	0.0605 (9)	
H7A	0.3343	0.5117	0.5803	0.073*	
H7B	0.4520	0.5342	0.5918	0.073*	
C8	0.3502 (2)	0.2801 (4)	0.50990 (13)	0.0491 (8)	
C9	0.3895 (2)	0.1729 (3)	0.46270 (12)	0.0426 (7)	
C10	0.3298 (2)	0.0857 (3)	0.42331 (13)	0.0478 (7)	
C11	0.3839 (3)	−0.0003 (4)	0.38152 (14)	0.0584 (9)	
H11A	0.3478	−0.0579	0.3539	0.070*	
C12	0.4897 (3)	−0.0013 (4)	0.38041 (15)	0.0615 (9)	
H12A	0.5255	−0.0579	0.3524	0.074*	
C13	0.5402 (2)	0.0838 (4)	0.42194 (13)	0.0576 (8)	
H13A	0.6116	0.0817	0.4219	0.069*	
C14	0.2149 (2)	0.0680 (4)	0.41832 (14)	0.0546 (8)	

### Atomic displacement parameters (Å^2^)

**Table:**

	*U*^11^	*U*^22^	*U*^33^	*U*^12^	*U*^13^	*U*^23^
N1	0.0415 (13)	0.0663 (17)	0.0410 (14)	0.0020 (13)	0.0024 (11)	−0.0069 (13)
O1	0.0363 (11)	0.1000 (16)	0.0595 (13)	0.0078 (11)	0.0056 (11)	−0.0135 (14)
C1	0.072 (2)	0.103 (3)	0.050 (2)	−0.014 (2)	−0.0011 (18)	−0.019 (2)
N2	0.0376 (12)	0.0689 (17)	0.0478 (15)	0.0004 (12)	0.0062 (11)	−0.0022 (15)
O2	0.0640 (15)	0.1065 (19)	0.0865 (18)	−0.0091 (15)	−0.0183 (14)	−0.0250 (18)
C2	0.097 (3)	0.155 (4)	0.045 (2)	0.000 (3)	−0.010 (2)	−0.016 (3)
O3	0.0432 (12)	0.0951 (18)	0.0701 (15)	0.0013 (12)	−0.0042 (11)	−0.0104 (15)
C3	0.121 (4)	0.115 (3)	0.043 (2)	0.011 (3)	0.014 (2)	0.002 (3)
C4	0.090 (3)	0.111 (3)	0.072 (3)	−0.003 (3)	0.022 (2)	0.017 (3)
C5	0.057 (2)	0.104 (3)	0.065 (2)	0.000 (2)	0.0027 (17)	0.010 (2)
C6	0.0551 (18)	0.063 (2)	0.0434 (18)	0.0046 (16)	0.0044 (15)	−0.0106 (17)
C7	0.061 (2)	0.066 (2)	0.054 (2)	0.0011 (16)	0.0074 (16)	−0.0053 (19)
C8	0.0407 (16)	0.064 (2)	0.0428 (17)	0.0004 (15)	0.0053 (14)	0.0043 (16)
C9	0.0425 (14)	0.0538 (17)	0.0316 (15)	−0.0007 (14)	0.0031 (12)	0.0031 (15)
C10	0.0486 (17)	0.0525 (17)	0.0425 (17)	0.0008 (14)	0.0033 (14)	0.0076 (17)
C11	0.068 (2)	0.069 (2)	0.0390 (17)	0.0004 (18)	0.0001 (16)	−0.0058 (19)
C12	0.064 (2)	0.070 (2)	0.050 (2)	0.0057 (18)	0.0082 (17)	−0.0099 (19)
C13	0.0458 (18)	0.071 (2)	0.0562 (19)	0.0061 (16)	0.0127 (16)	−0.0013 (19)
C14	0.0488 (19)	0.0655 (19)	0.0494 (19)	−0.0037 (17)	−0.0065 (15)	0.0055 (19)

### Geometric parameters (Å, º)

**Table:**

N1—C8	1.312 (3)	C4—C5	1.384 (5)
N1—C7	1.460 (4)	C4—H4A	0.9300
N1—H1A	0.8600	C5—C6	1.398 (5)
O1—C8	1.249 (3)	C5—H5A	0.9300
C1—C6	1.369 (4)	C6—C7	1.502 (4)
C1—C2	1.397 (5)	C7—H7A	0.9700
C1—H1B	0.9300	C7—H7B	0.9700
N2—C13	1.327 (4)	C8—C9	1.506 (4)
N2—C9	1.353 (3)	C9—C10	1.405 (4)
O2—C14	1.214 (4)	C10—C11	1.398 (4)
C2—C3	1.357 (5)	C10—C14	1.508 (4)
C2—H2B	0.9300	C11—C12	1.378 (4)
O3—C14	1.310 (4)	C11—H11A	0.9300
O3—H3B	0.8200	C12—C13	1.367 (4)
C3—C4	1.357 (5)	C12—H12A	0.9300
C3—H3A	0.9300	C13—H13A	0.9300
			
C8—N1—C7	123.4 (2)	C6—C7—H7A	108.8
C8—N1—H1A	118.3	N1—C7—H7B	108.8
C7—N1—H1A	118.3	C6—C7—H7B	108.8
C6—C1—C2	120.7 (4)	H7A—C7—H7B	107.7
C6—C1—H1B	119.7	O1—C8—N1	121.1 (3)
C2—C1—H1B	119.7	O1—C8—C9	123.3 (3)
C13—N2—C9	118.5 (3)	N1—C8—C9	115.6 (2)
C3—C2—C1	120.3 (4)	N2—C9—C10	122.5 (3)
C3—C2—H2B	119.9	N2—C9—C8	111.0 (2)
C1—C2—H2B	119.9	C10—C9—C8	126.5 (2)
C14—O3—H3B	109.5	C11—C10—C9	116.1 (3)
C2—C3—C4	120.0 (4)	C11—C10—C14	113.3 (3)
C2—C3—H3A	120.0	C9—C10—C14	130.6 (3)
C4—C3—H3A	120.0	C12—C11—C10	121.3 (3)
C3—C4—C5	120.7 (4)	C12—C11—H11A	119.3
C3—C4—H4A	119.6	C10—C11—H11A	119.3
C5—C4—H4A	119.6	C13—C12—C11	117.7 (3)
C4—C5—C6	120.1 (4)	C13—C12—H12A	121.2
C4—C5—H5A	119.9	C11—C12—H12A	121.1
C6—C5—H5A	119.9	N2—C13—C12	123.9 (3)
C1—C6—C5	118.2 (3)	N2—C13—H13A	118.1
C1—C6—C7	120.4 (3)	C12—C13—H13A	118.1
C5—C6—C7	121.4 (3)	O2—C14—O3	119.7 (3)
N1—C7—C6	113.9 (2)	O2—C14—C10	119.6 (3)
N1—C7—H7A	108.8	O3—C14—C10	120.7 (3)
			
C6—C1—C2—C3	−0.1 (7)	N1—C8—C9—N2	−2.3 (4)
C1—C2—C3—C4	0.9 (7)	O1—C8—C9—C10	−2.5 (5)
C2—C3—C4—C5	−0.4 (7)	N1—C8—C9—C10	177.7 (3)
C3—C4—C5—C6	−1.0 (6)	N2—C9—C10—C11	−2.8 (4)
C2—C1—C6—C5	−1.2 (6)	C8—C9—C10—C11	177.2 (3)
C2—C1—C6—C7	−179.2 (3)	N2—C9—C10—C14	177.0 (3)
C4—C5—C6—C1	1.7 (5)	C8—C9—C10—C14	−3.0 (5)
C4—C5—C6—C7	179.7 (3)	C9—C10—C11—C12	1.5 (4)
C8—N1—C7—C6	93.2 (4)	C14—C10—C11—C12	−178.4 (3)
C1—C6—C7—N1	102.4 (4)	C10—C11—C12—C13	0.5 (5)
C5—C6—C7—N1	−75.5 (4)	C9—N2—C13—C12	0.2 (5)
C7—N1—C8—O1	−1.8 (5)	C11—C12—C13—N2	−1.4 (5)
C7—N1—C8—C9	178.1 (2)	C11—C10—C14—O2	7.7 (4)
C13—N2—C9—C10	2.1 (4)	C9—C10—C14—O2	−172.1 (3)
C13—N2—C9—C8	−178.0 (3)	C11—C10—C14—O3	−174.0 (3)
O1—C8—C9—N2	177.6 (3)	C9—C10—C14—O3	6.2 (5)

### Hydrogen-bond geometry (Å, º)

**Table:**

*D*—H···*A*	*D*—H	H···*A*	*D*···*A*	*D*—H···*A*
N1—H1*A*···O3^i^	0.86	2.24	3.033 (3)	154
O3—H3*B*···O1	0.82	1.62	2.435 (3)	179

Symmetry code: (i) *x*+1/2, −*y*+1/2, −*z*+1.

## References

[bb1] Enraf–Nonius (1994). *CAD-4 EXPRESS* Enraf–Nonius, Delft, The Netherlands.

[bb2] Harms, K. & Wocadlo, S. (1995). *XCAD4* University of Marburg, Germany.

[bb3] Koch, C., Görls, H. & Westerhausen, M. (2008). *Acta Cryst.* E**64**, o2358. 10.1107/S1600536808037021 PMC2959924 21581331

[bb4] Konshin, M. E., Syropyatov, B. Y., Vakhrin, M. I., Neifel’d, P. G., Feshin, V. P., Shurov, S. N. & Odegova1, T. F. (2010). *Pharm. Chem. J.* **44**, 476–479.

[bb5] Sheldrick, G. M. (2008). *Acta Cryst.* A**64**, 112–122. 10.1107/S0108767307043930 18156677

